# Supplementary material for: Understanding of the Mechanism for Laser Ablation-Assisted Patterning of Graphene/ITO Double Layers: Role of Effective Thermal Energy Transfer
Source: Micromachines (Basel). 2020 Aug 29;11(9):821. doi: 10.3390/mi11090821 (PMC7570164; doi:10.3390/mi11090821)
Supplement: Supplementary file 1 [file micromachines-11-00821-s001.pdf]

Supplementary Materials: Understanding of the Mechanism for Laser Ablation-Assisted Patterning of Graphene/ITO Double Layers: Role of Effective Thermal Energy Transfer

Hyung Seok Ryu, Hong-Seok Kim, Daeyoon Kim, Sang Jun Lee, Wonjoon Choi, Sang Jik Kwon, Jae-Hee Han and Eou-Sik Cho

Simulations using the COMSOL MULTIPHYSICS software

For more in details, both PET substrates in the base of Figure 6a,c of the main text have square shapes (5 μm × 5 μm) with height of 0.5 μm. In case of ITO layers, the thicknesses were set to 7 nm above the PET substrates. In case of the graphene layer, whose actual thickness was 3 Å, we did not create a physical layer on top of the ITO film in Figure 6a. It was because the mesh geometry was hard to define in the graphene layer because of its extremely thin dimension compared to ITO layer and PET substrate. Even if the exceptionally fine mesh geometry were to be defined, it would take too much time and computational cost to obtain the calculation results. Therefore, we assumed the existence of single layered graphene by applying the 'Heat transfer in thin layer' boundary condition, which is provided by the COMSOL MULTIPHYSICS software. By applying this built-in boundary condition, we can assume the existence of 2D graphene layer on top of the ITO film, since this technique allows us to freely change the thermal property of the boundary layer of the open part of the ITO film. This mode assumes the thermal equilibrium between both sides of the layered shells (air side and ITO film side). Normally, this method is utilized when the thermal conductivity of the boundary layer is much higher than that of the surrounding base materials. Thus, we could simulate the effect of single layered graphene without considering its physical existence, thereby saving the computational cost of the simulation. Both simulations in Figure 6a,c were performed by using 'Heat transfer in solid' module on the software. The heat sources were set as the beam power distributions, which were assumed to have the Gaussian distribution with 99% of the total beam intensity within the preset beam radii. Laser irradiation durations were set to 10 ns to mimic the actual laser ablation process.

Table 1. Optical transmittance and sheet resistance of the ITO/PET and 1-layer-graphene/ITO/PET substrates.

	ITO on PET	1-layer-graphene/ITO on PET
Transmittance (400~800 nm) [%]	86.92	86.52
Sheet resistance (Ω/□) [%]	388.68	349.24

Table 2. The additional data for the optical transmittance, sheet resistance, and figure of merit (FOM) of the ITO/PET, 1-layer-graphene/ITO/PET, and 2-layer-graphene/ITO/PET substrates.

Measurement \ Samples	ITO on PET	ITO/ 1-graphene on PET	ITO/2-graphene on PET
Transmittance [%]	86.92	86.52	82.96
Sheet Resistance [Ω/□]	388.68	349.24	94.58
Figure of Merit [$10^{-4}Ω^{-1}$]	6.333	6.733	37.14

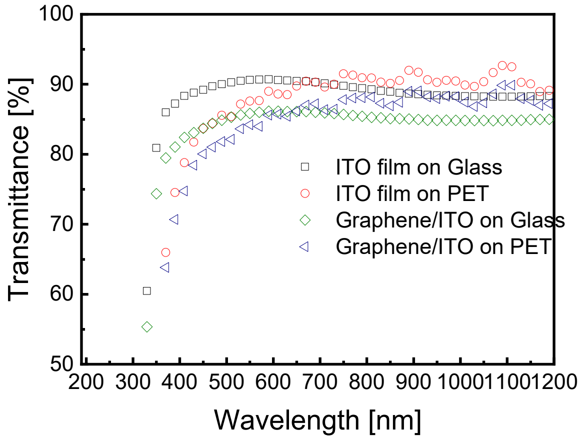

Figure S1. A plot for the optical transmittance as a function of wavelength of incident light of the UV-visible spectrometer for different substrates.

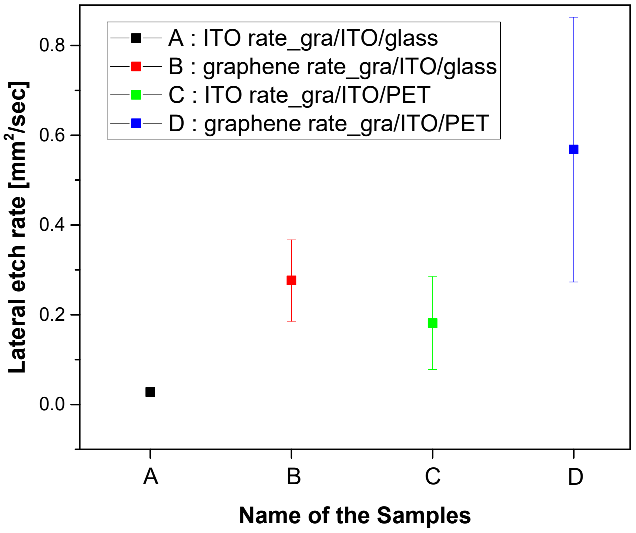

Figure S2. A plot for the etch rate in the lateral (or in-plane) direction for the different samples.
